# Novel Advances to Post-Stroke Aphasia Pharmacology and Rehabilitation

**DOI:** 10.3390/jcm10173778

**Published:** 2021-08-24

**Authors:** Natalia Cichon, Lidia Wlodarczyk, Joanna Saluk-Bijak, Michal Bijak, Justyna Redlicka, Leslaw Gorniak, Elzbieta Miller

**Affiliations:** 1 Biohazard Prevention Centre, Faculty of Biology and Environmental Protection, University of Lodz, Pomorska, 141/143, 90-236 Lodz, Poland; michal.bijak@biol.uni.lodz.pl (M.B.); leslaw.gorniak@biol.uni.lodz.pl (L.G.); 2 Department of Occupational Diseases and Environmental Health, Nofer Institute of Occupational Medicine, 91-348 Lodz, Poland; lidia.monika.wlodarczyk@gmail.com; 3 Department of General Biochemistry, Faculty of Biology and Environmental Protection, University of Lodz, Pomorska, 141/143, 90-236 Lodz, Poland; joanna.saluk@biol.uni.lodz.pl; 4 Department of Neurological Rehabilitation, Medical University of Lodz, Milionowa, 14, 93-113 Lodz, Poland; justyna.redlicka@umed.lodz.pl (J.R.); elzbieta.dorota.miller@umed.lodz.pl (E.M.)

**Keywords:** post-stroke aphasia, speech language therapies, cognitive neurorehabilitation, telerehabilitation, pharmacotherapy, physical medicine

## Abstract

Aphasia is one of the most common clinical features of functional impairment after a stroke. Approximately 21–40% of stroke patients sustain permanent aphasia, which progressively worsens one’s quality of life and rehabilitation outcomes. Post-stroke aphasia treatment strategies include speech language therapies, cognitive neurorehabilitation, telerehabilitation, computer-based management, experimental pharmacotherapy, and physical medicine. This review focuses on current evidence of the effectiveness of impairment-based aphasia therapies and communication-based therapies (as well as the timing and optimal treatment intensities for these interventions). Moreover, we present specific interventions, such as constraint-induced aphasia therapy (CIAT) and melodic intonation therapy (MIT). Accumulated data suggest that using transcranial magnetic stimulation (TMS) and transcranial direct current stimulation (tDCS) is safe and can be used to modulate cortical excitability. Therefore, we review clinical studies that present TMS and tDCS as (possible) promising therapies in speech and language recovery, stimulating neuroplasticity. Several drugs have been used in aphasia pharmacotherapy, but evidence from clinical studies suggest that only nootropic agents, donepezil and memantine, may improve the prognosis of aphasia. This article is an overview on the current state of knowledge related to post-stroke aphasia pharmacology, rehabilitation, and future trends.

## 1. Introduction

Aphasia is one of the most common clinical features of functional impairment after a stroke, affecting 21–40% of post-stroke patients. Aphasia is a language disorder with a broad clinical picture most often caused by damage to the dominant hemisphere of the brain [1]. Characteristics may include creating words from one’s own understanding of grammar, and motor speech impairment. Hence, aphasia can be defined as a language processing disorder at the morphological, phonological, syntactic, or lexical semantic levels. For language functioning, the left hemisphere is used by dextral individuals (99%) and 70% of sinistromanual individuals, the remaining 30% of left-handers use 15% of the right hemisphere and both hemispheres, respectively [2]. Impaired functional communication in aphasia patients can lead to functional deterioration, poor functional recovery, depression, and increased social isolation. 

In most cases, post-stroke aphasia (PSA) co-occurs with other cognitive-behavioral deficits, such as impaired perception, attention, or memory. The Boston Classification System, developed on the basis of the correlation of the clinical picture and radiological examinations, distinguishes the following syndromes of aphasia: Broca’s, Wernicke’s, anomic, transcortical motor, transcortical sensory, and conductive and global aphasia [3]. 

Spontaneous improvement of linguistic functions, depending on the location, size of the infarction, severity of the initial neurological deficits, as well as individual characteristics of the patient (the degree of hemispherical laterality for language functions, age and level of education), occurs, to some extent, within weeks or months after an ischemic event [4]. Recovery after stroke depends on the intensity of the neuroplasticity processes that begin immediately after an ischemia/reperfusion incident. The ischemic cascade causes changes to gene expression, leading to increased brain excitability, angiogenesis, increased concentration of growth factors, activation of synaptogenesis and neuritogenesis, and remodeling of axons. Importantly, molecular mechanisms of self-repair occur not only in the area of ischemia, but also in the ipsilesional region conjoined to the damaged area, contralateral hemispheres, and in the spinal cord. In most cases of aphasia, the most effective method of recovery involves the assumption the functions by the preserved left-hemispheric structures, adjacent to the damaged region [5]. Each stroke phase is characterized by the activation of different molecular mechanisms, allowing the preservation and/or restoration of the functions of the damaged area. The first two time windows are in the acute and subacute stroke phases, during which the most intense neuroplasticity processes take place [6]. The first time window to preserve the damaged tissue occurs a few hours after the stroke, and then neuroprotective and reperfusion treatments are the most desirable. The second important therapeutic period, during which neuron repair occurs spontaneously, exists days to weeks after a stroke. The third time window corresponds with the chronic stroke phase, in which endogenous repair takes place less dynamically. Nevertheless, appropriate therapeutic interventions allow for modifications of the functions and structure of the brain [5]. 

**Figure 1:**
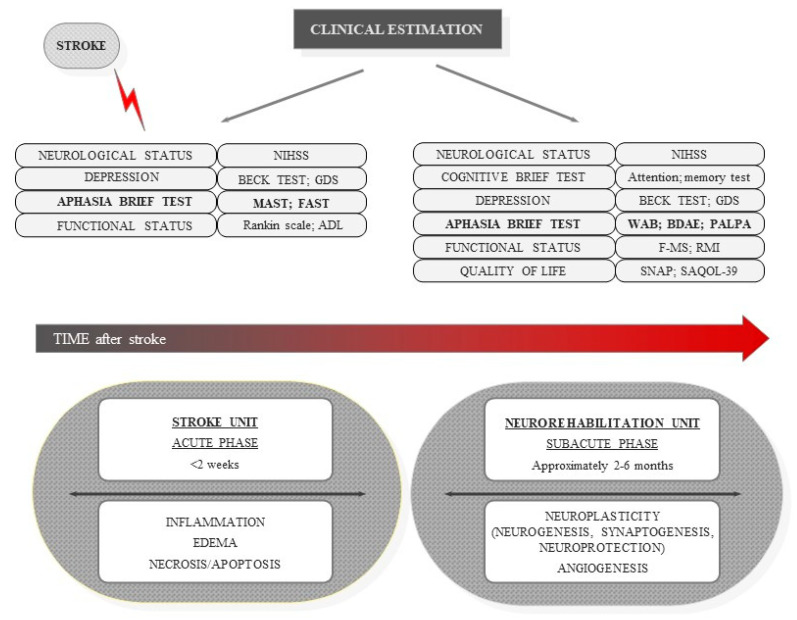
Diagram depicts brain stroke stages with clinical testing tools in different stroke phases evaluation. (NIHSS: National Institutes of Health Stroke Scale, GDS: geriatric depression scale, MAST: Mississippi Aphasia Screening Test, FAST: Frenchay Aphasia Screening Test, ADL: activity daily living, WAB: Western Aphasia Battery, BDAE: Boston Diagnostic Aphasia Examination, PALPA: Psycholinguistic Assessment of Language Processing in Aphasia, F-MS: Fugl-Meyer Scale, RMIL: Rivermead Mobility Index, SNAP: social network with aphasia profile test, SAQOL-39: aphasia quality of life scale-39).

The aim of rehabilitating patients with aphasia is to enable communication with the environment, compensate behavioral deficits manifested by changing the programs of a given activity, and improve the effectiveness of patient behavior (i.e., by modifying their environments, see Figure 1). The rehabilitation program should be determined, primarily, by a number of factors, including the severity and type of aphasia, etiology, other cognitive-behavioral dysfunctions accompanying aphasia, and stage of recovery [7]. The most commonly used clinical terminology to describe the recovery phases after a stroke are: acute (stroke unit), subacute (neurorehabilitation unit—active rehabilitation), and chronic (compensation rather than functional restoration). There is a lack of consensus on the duration of each phase. In general, the first 2–6 months after a stroke are defined as the early phase (acute and subacute phases) [8,9,10]. However, based on our clinical observations and studies, we suggest that the first 3 months—especially the first 6 weeks—are critical in post-stroke recovery. Significant improvement in language performance occurs within the first 2 weeks after a stroke, after which the dynamics of recovery slow down [4]. Generally, rehabilitation promotes early beginning of treatment due to interconnections between two processes; spontaneous and learning-dependent neural recovery. Taking all of the above into account, our review focuses on the each stage of post-stroke aphasia. This article emphasizes the significance of early treatment and the intervention objectives, which should be considered before starting therapy [10,11].

Currently, there is no single universally accepted algorithm for treating aphasia. All available therapies, without clear differences in their effectiveness, should be adjusted to the individual needs of the patients. This article is an overview of the current state of knowledge related to post-stroke aphasia pharmacology, rehabilitation, and future trends.

## 2. Assessment and Aphasia Outcomes

Aphasia tests are used to identify impaired language functions, assign these symptoms to specific types of aphasia, and indicate the depth of speech deficits. The tests should take into account the variability of symptoms of disorders, in connection with the time factor, and minimize the influence of intelligence, education, and memory in the assessment of aphasic disorders. The scales are also adjusted due to linguistic differences [12]. In regard to the use of metric tools in aphasia diagnosis—collecting diagnostic data involves a standardized procedure (one that is highly controlled). Interpretation of the test data should differentiate among healthy people, people with aphasia, and people without aphasia, but with symptoms of cerebral pathology [13]. A fairly large group of tools—used to study language disorders among patients with brain damage—are metric techniques, which are primarily used for screening. Screening tests can be used as techniques to create general diagnostic hypotheses about the occurrence of aphasia-type linguistic disorders in respondents, based on a quick quantitative assessment of the formation and understanding of statements, writing, reading, selected repetition, drawing, analysis, letter synthesis tests, etc. [12]. A precise assessment of the type and, in particular, the degree of aphasic speech disorder in the acute stroke period is difficult. Short scales are characterized by little diagnostic possibilities, while long scales are usually complicated, time-consuming, and tiring for patients. Table 1 shows the most popular scales. We compared the time of examination, tested language functions, identifiable types of aphasia, and criteria to determine the severity of speech deficits [14,15].

## 3. Methods

A literature search was conducted using MEDLINE and EMBASE databases. A total of 125 articles were analyzed, including 79 original research papers and 45 reviews (meta-analyses, systematic reviews, literature reviews). In this review, we primarily included articles from the last 10 years. Due to the limited number of randomized clinical trials available, minor trials with a small number of patients were also included in the study. All analyzed articles concerning acute post-stroke aphasia described both standard and innovative approaches to therapy and were published in English. Search terms included “post-stroke aphasia therapy”, “aphasia treatment after stroke”, “standard care of post-stroke aphasia”, “post-stroke aphasia rehabilitation”, and “post-stroke aphasia pharmacology”. Therefore, we excluded articles published in languages other than English. No restrictions were set for the type of stroke, disability level, or treatment strategy. Independently, three authors searched databases for articles on post-stroke rehabilitation/therapy, and three for pharmacology in post-stroke aphasia. 

## 4. Standard Care of Aphasia

Treatment of post-stroke patients with aphasia is based on strictly defined principles [7]. Different therapy objectives are distinguished, depending on the time of the infarction. In the acute/sub-acute phase, the therapy should focus on general speech stimulation, facilitating natural communication and providing emotional support to the patients. Then, it is crucial to establish emotional contact with the patients, encouraging them to maintain communication with their environments, as well as prevent inappropriate “compensation” of existing deficits. In the chronic phase, therapy should focus on developing and consolidating language skills and modifying communication behavior [16]. Primary and adjuvant therapies in the treatment of post-stroke aphasia are summarized in Table 2.

Speech and language therapy (SLT) is quintessential and the most common rehabilitation method for post-stroke patients with aphasia, with the primary goal of helping these patients communicate within their environments (i.e., in everyday life situations). Communication problems, in connection with cognitive impairment, can lead to depression, which in turn affects the patient’s recovery. Therefore, it is paramount to enable effective communication, both verbal and non-verbal, by any means [17]. 

SLT can be carried out in various forms. It has been shown that, compared to the lack of SLT, it has beneficial effects for patients, in regard to functional communication, expressive language, writing, and reading. The optimal intervention intensity and dose parameters in STL have not been determined so far. Some data suggest that high-intensity SLT over a shorter period of time has better results compared to low-frequency treatment over a longer period of time; however, research also shows that, more often, high-intensity SLT is discontinued by post-stroke patients [18]. Similarly, there is no consensus on the timing of initiation (and continuation) of rehabilitation after a stroke. The lack of reliable research on this subject does not clearly explain this problem. Although the timing of initiation of SLT has not been determined, it was suggested that if treatment is not conducted from the early stroke phase, then optimal benefits for the patient can be achieved in the chronic phase.

One SLT method is Multi-Modal Aphasia Therapy (M-MAT), which involves the use of all verbal and non-verbal strategies available to the patient, to increase the patient’s effectiveness of communicating with the environment. This method is mainly used in the treatment of severe motor aphasia and/or transcortical sensory aphasia [19]. In turn, augmentative and alternative communication (AAC) is based on non-verbal communication strategies due to the patient’s inability to communicate verbally. This method may be used temporarily during the early phase of a stroke when the aphasic disorder is most severe, or for a longer period during the chronic phase of a stroke, when language impairment is deeply established. AAC is used in severe aphasia, mainly in motor, but also in sensory aphasia [30]. The Intensive Comprehensive Aphasia Program (ICAP) is used in mild to moderate aphasias, from the subacute to the chronic phase of a stroke. ICAP consists of intensive exercises, individually adjusted to the disturbed functions, as well as exercises of speech functionality. This method uses a variety of techniques, including computer programs, psychoeducational techniques, and group activities. Rehabilitation is conducted five times a week, up to 3 h a day for a minimum of 2 weeks [20]. In turn, the progressive training of impaired linguistic functions related to the level of the patient’s clinical picture (semantic, phonological, syntactic, lexical, and motor speech realization) is characterized by Language Impairment-Based Therapy (LIBT). This technique is used in the treatment of various types of aphasia at each stage of the disease (from subacute to chronic) [21]. 

As previously mentioned, it is not possible to clearly determine which of the listed methods is the most effective. Numerous clinical studies only confirm the positive effects of all these methods compared to patients without SLT therapy; however, they do not indicate the superiority of one method over another [30,31,33,34,35,36,37]. The recovery mechanism related to SLT is not fully explained. The role of learning is also postulated—in addition to activating the spontaneous processes of self-repair and functional reorganization—although it is different than in the developmental period, during which incidental and implicit learning ensues. In post-stroke survivors, explicit learning occurs, i.e., the effortless reconstruction of linguistic knowledge through recalling grammatical structures, the meaning of concepts, repeating phrases and words, etc. Thus, the effects of the applied therapy also depend on the extent of the damage to the hippocampal system and its surrounding structures [38].

### 4.1. Constraint-Induced Aphasia Therapy (CIAT)

Constraint-induced aphasia therapy (CIAT) is a therapeutic method based on the need to communicate only with the use of language, without the use of non-verbal forms of communication. It was first developed by Pulvermuller et al. in 2001 [39], subsequently, it was modified into a new version called intensive language action therapy (ILAT), which is based on the three principles—massed practice, communicative relevance, and focusing of training the patient’s language possibilities and needs [40]. It is used in the treatment of aphasia with partially preserved expressive language skills, regardless of the stage of the stroke (from subacute to chronic). CIAT is a time-limited, intensive form of therapy that is conducted for 3–4 h a day for several days or weeks [22]. A clinical pilot trial to determine the efficacy of CIAT and conventional SLT therapy, with respect to linguistic expression in PSA, showed that reducing non-verbal forms improved functional communication. Importantly, the greatest improvement was noted when CIAT was conducted early in treatment and then traditional SLT was continued, compared to the reverse treatment regimen [35]. The meta-analysis conducted by Zhang et al., aimed at determining the most important components of CIAT therapy, did not observe greater effectiveness of the CIAT compared to other therapies. They noted that CIAT vs. other therapeutic programs showed no differences in repetition (0.08, 95% CI-11.88–12.03, *p* = 0.99), naming (3.97, 95% CI-7.86–15.79, *p* = 0.51), comprehension (−4.34, 95% CI-12.58–3.91, *p* = 0.30), and written language (−1.96, 95% CI-9.08–5.16, *p* = 0.59) [22]. In turn, a systematic review, comparing the effectiveness of CIAT with multimodal approaches to treatment, in which non-verbal forms are used in chronic aphasia, showed that there is no clear scientific evidence of the superiority of any of these methods. In this work, quality of life outcomes, impairment, and activity were assessed. A meta-analysis was impossible due to the methodological discrepancy and too few high-quality studies [41]. 

### 4.2. Cognitive Neurorehabilitation

Impaired cognitive functions, including attention, memory, learning, and visual–spatial orientation are also manifested in post-stroke symptoms of a focal brain injury, and occur in 53.4% of stroke survivors [42]. Often, a spontaneous reduction in cognitive impairment occurs in the subacute phase of a stroke, most often up in the third month after the onset of an ischemic event. However, there is a large group of patients with chronic progressive cognitive impairment [43,44]. Severe stroke, advanced age, and multifocal brain damage are negative prognostic factors concerning cognitive ability [45].

A posterior artery stroke, damaging the inferior medial parts of the temporal lobe, including the structures of the hippocampus, causes memory impairment. However, memory is a complex set of systems that cannot be tied to any specific brain structure [46]. Rehabilitation methods to reduce memory disorders are related to repetition, grouping information, creating new associations, checking memory traces, and remembering short therapeutic texts after a short time. One important method in regard to memory improvement is drawing therapy, in which the patient is supposed to remember and draw presented models. Another therapeutic option supporting semantic memory, as well as the organization of the obtained information, involves the patients collecting information about themselves, recreating their own lives on this basis [47]. A systematic review published by Cochrane Library in 2016 revealed that stroke survivors saw memory deficits improved shortly after cognitive therapy compared with the control group. However, there was no improvement in the independence in the daily functioning of patients. Importantly, no side effects of the therapies were observed [48].

Concentration problems in post-stroke patients are common. A patient’s inability to focus for a long period of time, being unable to concentrate on a specific task, and being easily distracted hamper the recovery process. Attention disorder neurotherapy is based on concentration exercises of increasing complexity, beginning with alertness exercises and gradually moving to more complex processes, such as selectivity and divisibility. This therapy can be conducted with the use of a computer, as well as paper-and-pencil exercises, and in the case of group therapy—with the use of verbal and non-verbal games [23]. A systematic review published by Cochrane Library aimed to determine the impact of cognitive neurorehabilitation on a patient’s ability to perform daily activities, concentration, quality of life, and mood. In the study, no evidence was found that cognitive rehabilitation improved general attention, mood, or quality of life in patients. Nevertheless, it was reported that patients who received cognitive therapy compared to the group that received standard post-stroke rehabilitation performed better on tasks where divided attention was required. Thus, it is necessary to conduct further research in order to reach unambiguous conclusions [24]. 

Currently, there are more reports on the relationship between cognitive function and linguistic ability. There are, presumably, common neuroanatomical–functional regions for both linguistic skills and components of executive functions. Therefore, damage of the structure for crucial importance for both abilities, linguistic and executive dysfunctions may occur in parallel. Executive functions enable the processes of initiating, planning, and monitoring behavior. Executive abilities require activity and coordination between the distributed regions of the brain and are associated primarily with neuronal activity within the frontal lobes, especially in the dorsolateral prefrontal cortex, but it has been suggested that the regulation of these functions depend on the interactions of cortical and subcortical structures [25,49]. Therapeutic methods supporting executive abilities consist of developing strategies and instructions, solving problems by defining them, and verifying the results. One important element of treatment is time management, which is trained by measuring the duration of exercise [50]. Nevertheless, all cognitive deficits are related, and neurocognitive therapy requires a comprehensive selection of exercises.

### 4.3. Telerehabilitation

Telerehabilitation is an important therapeutic tool for people who have limited access to conventional therapy, due to health, geographic, or financial reasons. The use of telerehabilitation is strongly recommended in the literature, due to an increase in the availability and effectiveness of therapy [26]. Information and communications technology enables personalized therapy via videoconferencing or telephone conversations and, thus, is a good alternative for patients with PSA. In addition, recently, the global epidemiological situation has generated interest in telemedicine, including telerehabilitation, often allowing the continuity of therapy [51,52,53]. Patients with PSA constitute a diverse population, therefore, when designing a therapeutic program using telerehabilitation, the skills and needs of patients should be consulted in order to eliminate all potential barriers related to technology [27].

Despite growing interest, there is little scientific evidence comparing telerehabilitation to face-to-face therapy. Most studies are described as ‘pilot’ studies with a small number of patients. In a randomized clinical trial of 21 patients with aphasia (after experiencing left hemispheric strokes), it was reported that telerehabilitation (eight treatment sessions) by videoconference showed similar efficacy in conversation assessment when compared to traditional therapy. Noteworthy, patients in the word-finding study group experienced better “picture naming” when compared to the control [52]. Similarly, in a study of 61 patients with PSA, results showed no difference in auditory understanding and naming between the telerehabilitation group and the control group (conventional therapy). Importantly, both groups experienced significant improvement in language functions [54]. In addition, it was shown that synchronous telespeech therapy improved functional communication in patients with PSA. This therapy resulted in the improvement and acceleration of conversation, as well as an increase in its effectiveness and the number of used communication strategies [51]. Two consecutive pilot studies, which included five and three patients with post-stroke anomia, respectively, also showed no differences between telerehabilitation and therapy “face-to-face” [55,56]. A study on the feasibility and acceptability of speech therapy in patients with PSA showed that over 93% of patients (n = 30) undergoing telerehabilitation via videoconferencing (1 h a day, five times a week for 4 weeks), in addition to standard treatment, rated the therapy as very good or good. Similar conclusions were noted in the opinions of speech therapists—two out of three were satisfied with the form of therapy. Technical errors were distinguished as one of the problems; however, this did not reduce satisfaction in the therapy [57]. The study results conducted so far indicate the effectiveness and feasibility of aphasia therapy in the form of videoconferences, however, it is necessary to conduct research on a larger number of patients. The results of the available evidence are promising and indicate the effectiveness and feasibility of videoconference therapy for aphasia. However, larger studies are needed to confirm the effects of telerehabilitation of PSA.

### 4.4. Computer-Based Management

The best clinical effects in the treatment of aphasia are as a result of intensive and long-term therapy. However, due to the high costs of long-term health care associated with the provision of a large amount of conventional speech therapy, problems with the availability of specialists in smaller towns, and often strong willingness of patients to increase the amount of training, computer-based management is eagerly used as a tool for the treatment of PSA [29]. Moreover, the advantages of speech therapy applications include the variety of short and long-term therapy, low costs, and effectiveness, but also enable therapy, not only under the supervision of speech therapists, but also at home under the supervision of people from the patient’s immediate surroundings [28].

A multicenter, single-blinded, randomized controlled clinical trial included 818 patients with PSA, who were divided into three groups: the test group undergoing standard care with self-managed computerized SLT, the control group subjected to standard care, and the control group with standard care and attention control. In this study, it was shown that computer-based therapy significantly improved word-finding skills, but did not affect functional conversation [58]. A systematic review by Zheng et al. has shown that computer-based therapy in patients with aphasia is an effective method of SLT therapy compared to no therapy, and shows potentially similar efficacy to treatment performed by specialists. However, this work included a small number of randomized trials, therefore the quality of the evidence is not high [59]. 

A characteristic feature of computer-based therapy is the possibility of conducting therapy both at home and in specialized centers. Importantly, patients with communication problems often withdraw from verbal contact and avoid activities that cause difficulties. Thus, the therapy based on multimedia applications enables patients to practice on their own, in comfortable conditions that provide a sense of security. One significant problem of the applications used in SLT is their “universality”, i.e., suitability for both adults and children, which is associated with an infantile interface and vocabulary. After suffering from a stroke, patients can experience discouragement, or depressive disorders can be aggravated, as although most of these patients have communication disorders, their intellectual abilities are preserved. Nevertheless, therapy based on multimedia applications has great therapeutic potential, in particular with regard to the treatment of chronic aphasia.

### 4.5. Melodic Intonation Therapy (MIT)

Melodic Intonation Therapy (MIT) emphasizes the prosody of speech by using extra-linguistic features of a spoken language, such as intonation, rhythm, and emphasis. Initially, communication is based on excessive rhythmic and/or melodious speaking, which is gradually replaced by natural speech prosody. This treatment method is used in all phases of the stroke, mainly in non-fluent Broca’s aphasia, most often in patients with left hemispheric ischemic damage. This technique aims to engage the right cerebral hemisphere, in particular the regions contralateral to the Broca’s area (pars triangularis) and the sensorimotor region, by tapping rhythmically with the left hand, which helps to better control mouth movements. In the majority of patients, the areas of the brain in the left hemisphere are responsible for speech, while areas responsible for singing are found in the right hemisphere; therefore, MIT therapy helps to reduce dependence on the left hemisphere [31]. 

A randomized, multicenter clinical trial evaluated the efficacy of MIT (n = 27) as well as the delay in initiation of therapy (control group, receiving MIT after 6 weeks after the study group) in patients in the sub-acute phase of a stroke (2–3 months after the ischemic episode) with non-fluent aphasia. It was shown that MIT, compared to the control group, significantly improved functional communication, as well as repetition of practiced material, and a delay in therapy resulted in a reduction in the improvement of repetition of trained items [32]. Then, in another study (n = 17), the investigators assessed the effects of MIT in patients with chronic PSA (>1 year after ischemic episode), the study group and the control group were similar to the first study (study group—MIT for 6 weeks; control—MIT after 6 weeks). This study showed that MIT improved the repetition of trained material, but had no effect on functional communication and repetition of untrained items. Thus, the authors suggest that MIT may be a more useful therapeutic tool in the sub-acute phase than in the chronic phase of a stroke [60]. In turn, Haro-Martínez et al. presented a pilot clinical trial (n = 20), evaluating the effect of MIT on the improvement of language functions in patients with non-fluent or global aphasia. The study included patients after unilateral left hemispheric stroke (<6 months after the ischemic event), in whom no damage was observed in the right hemisphere. The participants were divided into two groups: the research group received MIT from the start of treatment for 6 weeks, the control group received MIT with a 2–3-month delay. In this study, it was observed that MIT may have a beneficial effect on functional communication. However, no differences in functional outcomes were observed in patients who received MIT late [31]. Thus, further clinical trials with higher-quality scientific evidence in larger numbers of participants are needed to confirm these preliminary results.

## 5. Pharmacotherapy as an SLT Enhancer

The main purpose of pharmacological intervention in PSA is to improve and facilitate neuroplasticity, but evidence advocating pharmacological stimulation of neuroplasticity is limited. Despite little evidence, it is suggested that modulating the activity of neurotransmitter systems with pharmacological treatment is a promising strategy to recover language and communication deficits in PSA. As seen in a summary of pharmacological interventions in PSA (Table 3), there is quite a rich history of studies associated with this item. However, the most important challenge in exploring the area of aphasia recovery is the absence of animal models in regard to language, and limited clinical study data. 

In order to better understand the purpose and mechanism of action of drugs that support the treatment of aphasia, it is important to comprehend the physiological processes of language. Due to the fact that language is not a single cognitive function, different areas of the brain, interconnected with each other, are involved in speech functions [61]. According to the classic model of language organization, motor foci exist in Broca’s area (inferior frontal gyrus), whereas sensory language foci are located in Wernicke’s area (superior temporal gyrus), and arcuate fasciculus (connections between these areas) are enabled for auditory–motor interaction. The above theory has been postulated for a decade, but the use of advanced mapping and neuroimaging techniques has made it possible to understand, in more detail, the neuroanatomical functions of language. Currently a dual-stream language model has been identified; this is a network-based model of speech processing, including interconnected and parallel streams of subcortical and cortical regions. This model distinguishes the “dorsal” pathway that is responsible for phonological processing, running from the posterosuperior temporal to the inferior frontal cortices, and the “ventral” pathway participating in semantic processing, from the temporal pole to the basal occipitotemporal cortex, with anterior connections [62]. Among the neurochemical foundations of speech, an important role is assigned to the dopaminergic transmission through the nigrostriatal pathway. The release of striated dopamine from the ventral tegmental area (mesocortical system) and the ventral portion of the pars compacta of substantia nigra (nigrostriatal system) results in speech generation. Moreover, it is believed that during speech production, dopamine may regulate the activity of the laryngeal motor cortex as well as other parts of the ganglia circuitry of the vocal basal. Thus, the mesocortical dopaminergic system (innervating the frontal-parietal network) and nigrostriatal system, both related to the modulation of syntactic language processes, and the social brain network, responsible for the pragmatic aspect of language functions, is responsible for the main linguistic processes [63,64]. 

There are few, well-estimated drugs in clinical trials. Those with the most significant data that suggest improvement in the prognosis of PSA are donepezil and memantine [65]. Donepezil, which reversibly inhibits the cholinesterases and, consequently, increases acetylcholine concentrations, is widely known for improving the cognitive functioning in Alzheimer’s disease patients. In a well prepared, randomized, controlled trial of Berthier et al., the utility of cholinesterase therapy for stroke-related aphasia was evaluated. The investigators showed a significant improvement with several clinical tests (Western Aphasia Battery, the Communicative Activity Log, and the Psycholinguistic Assessment of Language Processing) in the donepezil group (16 weeks, up to 10 mg daily). However, the therapeutic effect was not persistent after the end of treatment in week 20, suggesting that the advantages of donepezil are not associated with neural reorganization [66]. On the other hand, Woodhead et al., in a double-blind, placebo-controlled, crossover study, found that donepezil was not beneficial in improving PSA; it in fact had a negative influence on speech outcome [67]. Considering the statements by Zhang—that donepezil has an effect on improving the ability of oral expression, auditory comprehension, naming, and repetition—it should be considered a potential therapy in following studies [65]. 

There is also solid evidence of memantine efficacy in PSA treatment. The most significant improvement was observed in language modalities as repetition, naming, and spontaneous speech [65]. Memantine is a non-competitive NMDA-receptor antagonist, and like donepezil, it is approved for Alzheimer’s disease. In a double-blind, randomized placebo-controlled trial of Berthier et al. [68] memantine and CIAT effect on chronic post stroke aphasia were studied. The memantine group (20 mg daily) showed significantly better improvement on WAB score compared with the placebo group while the drug was taken (week 16, *p* = 0.002; week 18, *p* = 0.0001; week 20, *p* = 0.005) and at the washout assessment (*p* = 0.041). Moreover, better outcomes were reached combining memantine with CIAT and its beneficial effects were maintained in the long-term follow-up evaluation. Very interesting analysis of this study was performed by Barbancho et al. [69], where the functional brain correlates of language processing using event-related potentials (ERP) was evaluated. Results showed that aphasia regaining induced by memantine and CIAT is indexed by bilateral cortical potentials. 

There are several studies that have examined the role of amphetamine AMPH (which acts by promoting monoamines’ synaptic concentration) on language functioning in aphasic patients. Amphetamine’s mechanism of action focuses on dopamine and norepinephrine reuptake inhibition, but in animal studies, it was also observed to augment post-infarction neural sprouting and synaptogenesis. The positive effect of AMPH was observed in studies by Walker-Batson, where a majority of active subjects demonstrated improvement: 83% for D-amphetamine vs. 22% for placebo on the Porch Index of Communicative Ability (PICA) scale [70]. Another study that confirmed the positive effect of dextroamphetamine was conducted by Keser, where 10 subjects with chronic, non-fluent aphasia received dextroamphetamine or a placebo, along with transcranial direct current stimulation and SLT. A statistically-significant increase was found in the active experiment [71]. In a small study, Whiting also found AMPH beneficial for PSA treatment [72]. In contrast, in McNeil’s crossover, multiple-baseline study, there was no improvement after D-amphetamine or selegiline usage, with and without lexical-semantic activation inhibition therapy (L-SAIT), in two patients with chronic aphasia from stroke [73].

Piracetam is another drug that we considered; it is a derivative of gamma-aminobutyric acid (GABA), marketed as a treatment for myoclonus and as a cognitive enhancer. Piracetam facilitates cholinergic and excitatory amine neurotransmission, it is claimed to improve learning and memory. The data on piracetam for aphasia are, of course, varied as in all previously described drugs. In a large (n = 927), multicenter trial by De Deyn, there was no significant effect on the primary outcome measures of neurological status (including assessment of aphasia) after treating all acute stage stroke patients with piracetam [74]. However, post-hoc analysis of aphasic patients (n = 373) found that aphasia recovery after 12 weeks was better in the piracetam group vs. the control [75,76]. Similar results were identified by Kessler in chronic aphasia patients [77].

Dopaminergic strategies were also highly studied for stroke-related aphasia recovery. Bromocriptine, a D2 receptor agonist, and levodopa, a dopamine precursor (with peripheral decarboxylase inhibitor), are considered beneficial for aphasia treatment. In the study by Seniów, 39 patients with subacute strokes were randomized to receive either 100 mg levodopa or a placebo. Drug therapy continued for 3 weeks with concomitant SLT therapy. Improvement in all clinical metrics was found, but only repetition of phrases, sentences, words, and verbal fluency reached statistical significance [78]. Although the above-mentioned study indicates that levodopa may facilitate language therapy, Breitenstein et al. found it did not augment outcomes of high-intensity language therapy (63.8% for levodopa versus 66.5% for placebo) [79]. Leemann et al. also observed no advantage in levodopa versus the placebo in the subacute period after a stroke, when it was combined with intensive computer therapy [80]. Bromocriptine also appears to raise vain hopes, as there are several studies denying improvement in the treatment of aphasia after a stroke [65,81,82,83]. Nevertheless, in a study by Bragoni, a high dose of bromocriptine (up to 30 mg) was applied to 11 chronic non-fluent aphasics and a statistically significant benefit on reading comprehension was sustained, after a 60-day washout of the drug [84].

There are isolated reports about the potential profitable effects of other pharmacological treatments, such as zolpidem—a short-acting nonbenzodiazepine hypnotic, but case reports [85,86] show it requires further study. Other promising items are galantamine, atomoxetine, intranasal desmopressin, propranolol, and citicoline, but data regarding them in the treatment of aphasia after stroke are limited and inconclusive (more clinical study data are needed). Clinical trials reported that selective serotonin reuptake inhibitors (SSRIs), used within 3 months after a stroke, improved motor recovery, compared to the placebo [87], presenting better results in cognitive tasks [88]. Therefore, there was a hypothesis that SSRIs could be effective by potentiating the effects of speech therapy. However, a new, large, randomized study (EFFECTS) of 1500 acute post-stroke patients from 35 Swedish hospitals reported that the antidepressant fluoxetine (20 mg/day/6 months) had no impact on post-stroke recovery [89].

One noteworthy fact is that pharmacological manipulations may also worsen or delay language recovery. It is suggested that haloperidol, diuretics (for example hydrochlorothiazide), and topiramate are associated with post-stroke language disturbances.

## 6. Non-Invasive Brain Stimulation (NIBS) as an SLT Enhancer

In aphasia therapy, SLT and pharmacological treatments remain the “gold standard”, but other forms of adjuvant therapy are desirable in order to maximize the restorative capacity of the brain and enhance recovery, notably in the chronic phase of a stroke. NIBS techniques relevant to the treatment of aphasia include transcranial magnetic stimulations (TMSs) and transcranial direct current stimulations (tDCSs) (Table 4). Numerous studies have shown the improvement of linguistic functions in patients with aphasia, as well as the activation of neuroplasticity, confirming the effectiveness of this strategy [99].

### 6.1. Transcranial Magnetic Stimulation (TMS) 

Transcranial magnetic stimulation is a non-invasive and painless form of therapy, widely used in the neurorehabilitation in post-stroke patients [100,101,102,104]. TMS is based on generating a magnetic pulse that induces power in various regions of the cerebral cortex. Depending on the type of stimulation, one can distinguish between stimulating the cortex with a single TMS impulse and applying several impulses in short intervals—repetitive transcranial magnetic stimulation (rTMS). The rTMS procedure consists of regularly applied pulses of a given frequency within approximately 15 min. Depending on the frequency used, there are low-frequency stimulations (<1 Hz), which inhibit cortical excitability, and high-frequency stimulations (>1 Hz), which increase cortical excitability. Side effects of TMS therapy are uncommon and rather transient, including mild headaches and lightheadedness, seizures, syncope, scalp pain, neck pain, tingling, facial twitching, sleepiness and, cognitive/neuropsychological changes [105]. The aim of aphasia treatment by rTMS is to equiponderate the excitability of both hemispheres, and to realign the linguistic network. Under physiological conditions, the language areas are placed in the dominant hemisphere, but it was observed that, in aphasia patients realizing language tasks, the perilesional regions of the left hemisphere, as well as homotopic areas, are characterized by abnormal excitability [106]. 

The clinical use of rTMS in the treatment of PSA has been intensively researched. In clinical trials, right IFG, right Broca’s area homolog, right Broca’s area homolog (1 Hz), then left Broca’s area (20 Hz), and right pars triangularis were administrated to rTMS [107]. A recent meta-analysis, which only included randomized control trials, aimed to evaluate the effectiveness of rTMS (of different frequencies) compared to sham stimulation, in post-stroke patients with aphasia. Treatment effects were assessed based on improved repetition, naming, and comprehension scores. It was observed that, in patients after rTMS therapy, with both low- and high-frequency, there was a significant improvement in naming (0.76, 95% CI 0.16–1.36, *p* < 0.001), while understanding and repetition did not change. Importantly, the rehabilitation of low-frequency TMS has significant short-term importance in the subacute phase of a stroke [108]. Moreover, the effectiveness of the use of low-frequency TMS in the rehabilitation of PSA was confirmed in a meta-analysis conducted by Li et al. It was shown that rTMS significantly improved the naming (0.51, 95% CI 0.16–0.68, *p* = 0.004) and modulated brain excitability (7.6 ± 33.55, 95% CI 10.7–26.20, *p* = 0.023), while comprehension and repetition did not change. Importantly, no side effects were noted [109]. In addition, Bacur and Papagano conducted a meta-analysis to determine the efficacy and safety of TMS in patients with PSA in the long-term. They found that rTMS has a moderate to large therapeutic effect in improving naming, and is sustained over time, in patients with aphasia; moreover, it is suitable for both the subacute and acute phases of a stroke [110]. Numerous studies indicate the validity of rTMS in the treatment of PSA, as it primarily improves naming, but also helps in remodeling linguistic networks in the left hemisphere. Nevertheless, further studies on large cohorts are necessary to determine the optimal treatment protocols and to take into account individual variability.

### 6.2. Transcranial Direct Current Stimulation (tDCS)

Transcranial direct current stimulation (tDCS) is a non-invasive and safe therapeutic technique for stimulating the brain. A one-time stimulation typically lasts 10–30 min; during this period, stimulus is delivered with a DC stimulus of 1–2 mA. This enables the polarization of the cell membranes of neurons, increasing or decreasing the level of cortical excitation. The nature of the induced cortical lesion depends on the electrode pole: the anode depolarizes the cell membrane of neurons, thereby enhancing the activity of the neural cortex. On the other hand, cathodic stimulation causes hyperpolarization of the cell membrane of neurons, leading to a decrease in cortical excitation [103,111]. In the subacute phase of a stroke, increased activation of the contralateral hemisphere and hypoactivation of the damaged hemisphere are observed. In minor ischemic injuries, in this phase, brain activity may normalize, leading to spontaneous self-repair. On the other hand, in extensive stroke injuries, the pattern of hypoactivation of the damaged hemisphere and hyperactivation of the healthy hemisphere are preserved, and the prolonged disproportion of the relative levels of hemispheric activation additionally disrupt the activity of damaged neural circuits and enhance the dysfunction, leading to inhibition of self-repair in the damaged region of the brain. Reduction of the tonic activation differences between the cerebral hemispheres can be achieved by anodic stimulation (a-tDCS), to increase the excitability of the injured hemisphere and/or cathodic stimulation (k-tDCS) and to reduce the excitation level of the contralateral hemisphere [112]. Mild transient skin erythema is common during tDCS, but generally, the use of tDCS is considered a minimal risk therapy [113].

tDCS and TMS are techniques that support SLT therapy in the recovery of patients with PSA. Small clinical pilot trials provided evidence for the efficacy and safety of this method in post-stroke rehabilitation [114,115,116,117,118]. The published meta-analyses, however, due to the low quality of scientific data, did not provide sufficient evidence of the effectiveness of tDCS (k-tDCS, a-tDCS, and dual-tDCS) in improving functional communication in PSA patients, compared to the sham exposure. Nonetheless, this therapy positively influences naming [119,120,121,122]. Left IFG, right Broca’s homolog, left primary motor cortex, area of the greatest left hemisphere activation, left inferior frontal cortex, right cerebellum, bilateral stimulation of left IFG and right IFG, and the left dorsolateral prefrontal cortex are brain regions that have been administered by tDCS in clinical trials [107]. 

A meta-analysis presented by the Cochrane Library, which included 21 randomized clinical trials, showed no evidence of improved functional communication both after tDCS therapy (0.17, 95% CI-0.20–0.55, *p* = 0,37), and during follow-up (0.14, 95% CI-0.31–0.58, *p* = 0.55). However, tDCS improved noun-naming after therapy (0.42, 95% CI 0.19–0.66, *p* = 0.0005), and during therapy (0.87, 95% CI 0.25–1.48, *p* = 0.006), but it did not improve verb naming. There were also no adverse effects and no effect of tDCS on cognitive functioning [120]. Nevertheless, a meta-analysis by Elsner et al., which aimed to determine the efficacy and safety of different stimulation subtypes (k-tDCS, a-tDCS, and dual-tDCS), showed relative efficacy in improving naming (without affecting functional communication) as a result of tDCS, and the most effective was the anodic stimulation (0.51, 95% CI 0.11–0.90), in particular of the left inferior frontal gyrus (IFG) [119]. The positive effect of tDCS on naming (endpoint) and greater effectiveness of a-tDSC compared to k-tDCS (*p* = 0.004) were also noted in the meta-analysis presented by Rosso et al. (0.8, 95% CI 0.27–1.33, *p* = 0.002). Importantly, the endpoint was dose-dependent, greater after left-hemispheric stimulation compared to right-hemispheric stimulation (*p* = 0.005), and equally favorable regardless of the severity of aphasia and post-stroke delays [121]. Moreover, it was noted that the most favorable effects of PSA treatment with tDCS are observed in the chronic phase of the disease [110,122,123]. In order to unequivocally determine the effectiveness of the intervention, further randomized clinical trials with larger group sizes are necessary.

## 7. Limitation/Discussion

Most of these studies are characterized by a lack of sufficient numbers of double-blind, placebo-controlled trials, with large sample sizes. Pharmacological treatments show a low level of effectiveness and a lack of knowledge about their mechanisms of action in the human body. Moreover, side effects during long-term treatment were observed. There are many problems with conducting RCTs that are currently considered as major standards for unbiased research. However, the PWA groups are heterogeneous and post-stroke aphasia is characterized by an unstable status, with rapid changes, and often significant improvement in the very early phase (<2 weeks after stroke) [4,7]. Therefore, it is difficult to form adequate subgroups and estimate the impact of therapy on patient improvement. An important problem in most clinical studies is the lack of differentiation in terms of aphasia subtypes (Global, Wernicke’s, or Broca’s aphasia), resulting in a lack of comparison between subtypes and, more importantly, a lack of recommendations in using proper therapy in a particular aphasia subtype. Therefore, it is not possible to estimate the precise effectiveness of treatment [107]. Future trials should be created to fulfil the purposes of stratified analyses; sample sizes ought to be large, preferably multicenter, or cross-national. 

## 8. General Conclusions

This review provided a comprehensive overview of novel approaches to pharmacology and rehabilitation of aphasia in the early stage of post-stroke treatment, which may facilitate future decisions in patient therapy and trigger future innovative clinical studies.

Currently, the most common treatment used in clinical practice is combined conventional speech therapy concomitant with innovative treatment [107]. Traditional speech and language therapy is effectively used for speech and language improvement. However, virtual reality (VR) is a promising tool that could be used as a self-operative, intensive treatment at a patient’s home, even for several hours a day. VR can realize a patient’s everyday activities; thus, it can also enhance cognitive functioning [124]. Currently, a promising therapy that can enhance traditional SLT is NIBS. Both of these techniques, tDCS and repetitive TMS (rTMS), are not only used in research in vivo, but also in clinical practice [107]. Data suggest the (potential) high-effectiveness of the mixed approach in motor recovery and the positive effects of VR concomitant with tDCS [125,126,127] or telerehabilitation [128]. Moreover, tDCS might be one of the most promising therapies used in additional to language training; it is cost-effective, safe, with low adverse effects. A recent study that combined dextroamphetamine therapy with tDCS and language therapy was positively investigated by Keser et al. [71].

The bases for recovery in patients with post-stroke aphasia, in the first stage of the disease, are the various self-repair processes that weaken over time. An interdisciplinary team, including a doctor, speech therapist, and neuropsychologist, must be involved in the process of linguistic restoration. Aphasic disorders affect the entire healing process, the basis of which is speech and language therapy aimed at compensating and/or restoring disturbed functions, modulating and supporting the entire process. Importantly, other therapeutic strategies, such as pharmacotherapy or neurostimulation, require methodologically rigorous clinical trials to determine their effectiveness. Most clinical trials are conducted in extremely small groups, so the quality of the research evidence is moderate or low. Despite the limited amount of scientific evidence, there is no doubt that the treatment of PSA is beneficial for patients; it should be individualized and conducted from an early stage, and in the long-term, until satisfactory results are achieved.

## Figures and Tables

**Table 1:** A set of standard tests to assess aphasia overcomes.

Scale	Time of Examination	Tested Language Functions	Identifiable Types of Aphasia
FAST (The Frenchay Aphasia Screening Test)	5–10 min	understanding spoken language production, reading, writing	presence of aphasia
MAST (The Mississippi Aphasia Screening Test)	5–15 min	understanding spoken language production, reading, writing	presence of aphasia
BDAE (Boston Diagnostic Aphasia Examination)	20–45 min (for the shortened version) 2–6 h (for the extended version)	understanding spoken language production, reading, writing	all types of aphasia: Broca’s aphasia, Wernicke’s aphasia, anomic aphasia, global aphasia, isolation, transcortical motor aphasia, transcortical sensory aphasia, conduction aphasia
WAB (The Western Aphasia Battery)	30–60 min	understanding spoken language production, reading, writing	all types of aphasia
PALPA (The Psycholinguistic Assessments of Language Processing)	difficult to estimate, depends on the choice of subtest	understanding reading, writing	assess language processing abilities in aphasic patients
BNT (The Boston Naming Test)	20 min	picture naming	all types of aphasia
TOKEN TEST	10–30 min	understanding	all types of aphasia
CAT (The Comprehensive Aphasia Test)	90–120 min	understanding spoken language production, reading, writing	all types of aphasia

**Table 2:** The main and adjuvant therapies in post-stroke aphasia.

Methods		Major Characteristics	Advantages	Limitations	
**SLT (Speech and language therapy)**	Conventional SLT	Facilitating communication with the environment in everyday life situations.	The most common rehabilitation method of PSA. If treatment is not conducted from the early phase of the stroke, then optimal benefits for the patient can be achieved in the chronic phase.	The optimal intensity and dose of STL has not been determined. There is no consensus on the timing of treatment initiation and its continuation.	[17,18]
	**M-MAT** (Multi-Modal Aphasia Therapy)	The use of all verbal and non-verbal strategies available to the patient to increase the effectiveness of communicating with the environment.	Applied in the treatment of severe motor aphasia and/or transcortical sensory aphasia.	The need for further clinical trials on a larger group of PSA.	[19]
	**ICAP** (The Intensive Comprehensive Aphasia Program)	Applied in mild to moderate aphasias, from the subacute to the chronic stroke phase.	Intensive exercises individually adjusted to the disturbed functions, as well as exercises of speech functionality. Variety of techniques, including computer programs, psychoeducational techniques, and group activities.	The need for further clinical trials on a larger group of PSA.	[20]
	**LIBT** (Language Impairment-Based Therapy)	Progressive training of impaired linguistic functions related to the level depending on the patient’s clinical picture (semantic, phonological, syntactic, lexical, and motor speech realization).	Applied in the treatment of various types of aphasia in each stage of the disease (from subacute to chronic).	The need for further clinical trials on a larger group of PSA.	[21]
	**CIAT** (Constraint-induced aphasia therapy)	Communicating only with the use of language, without the use of non-verbal forms of communication. Time-limited, intensive form of therapy that is conducted for 3–4 h a day for several days or weeks.	Applied in the treatment of aphasia with partially preserved expressive language skills, regardless of the stage of the stroke (from subacute to chronic).	The need for further clinical trials on a larger group of PSA.	[22]
**Cognitive neurorehabilitation**		Cognitive disorders, in particular memory and concentration disorders, are related to language functions.	A beneficial effect on the independence of the PSA.		[23,24,25]
**Telerehabilitation**		Using videoconference or telephone conversation in PSA therapy.	Important therapeutic tool for people who have limited access to conventional therapy for health; geographic or financial reasons. Strongly recommended in the literature, due to the increase in both the availability and effectiveness of therapy.	Designing a therapeutic program using telerehabilitation requires consulting the skills and needs of PSA in order to eliminate all potential barriers related to technology.	[26,27]
**Computer based management**		The use of IT tools to conduct PSA therapy.	Variety of short- and long-term therapy. Low costs and effectiveness. Enabling therapy not only under the supervision of speech therapists, but also at home, under the supervision of people from the immediate surroundings.	The need for further clinical trials on a larger group of PSA.	[28,29]
**AAC** (Augmentative and Alternative Communication)		Non-verbal communication strategies due to the inability to communicate verbally.	Applied temporarily during the early stroke when the aphasic disorder is most severe, or for a longer period during the chronic stroke phase, when the language impairment is deeply established. Used in severe aphasia, mainly in motor, but also in sensory aphasia.	The need for further clinical trials on a larger group of PSA.	[30]
**MIT** (melodic intonation therapy)		Main emphasis on the prosody of speech by using the extra-linguistic features of spoken language, such as intonation, rhythm and emphasis	Applied in all phases of stroke, mainly in non-fluent Broca's aphasia, most often in patients with left hemispheric ischemic damage Reducing left hemisphere dependence by involving the right cerebral hemisphere, in particular pars traingularis and the sensorimotor region by tapping rhythmically with the left hand, which helps to better control mouth movements	The need for further clinical trials on a larger group of PSA	[31,32]

**Table 3:** Summary of pharmacological intervention in post-stroke aphasia (PSA).

Drug	Positive Effect on PSA Observed	Negative or No Positive Effect on PSA Observed	Adverse Effects
DONEPEZIL	Zhang et al.; meta-analysis of 5 studies with 277 patient [65] Berthier et al.; randomized controlled trial; n = 13; chronic stage, dose up to 10 mg; period 16 weeks [66]	Woodhead et al.; baseline-controlled, crossover study, n = 20; chronic stage, period 5 weeks [67]	Insomnia Irritability Headaches Tiredness Dizziness Muscle cramps Increased sexual drive
MEMANTINE	Berthier et al.; randomized, double-blind, placebo-controlled, n = 28; dose 20 mg; period 16 weeks; chronic stage [68] Barbarancho et al.; randomized, double-blinded study, n = 27; dose, period, chronic stage [69]		
AMPHETAMINE	Keser et al.; randomized study, n = 10, period 1 day, chronic stage [71] Walker-Batson et al.; prospective, double-blinded study, n = 21; dose:10 mg; period 5 weeks; subacute stroke [70] Whiting et al.; double-blind, placebo-controlled, crossover design; n = 2, chronic stage [90]	McNeil et al.; crossover, multiple-baseline study; n = 2, chronic stage [73]	Insomnia
LEVODOPA	Seniów et al.; randomized study; n = 39; dose: 5 × 100 mg; period: 3 weeks; subacute stroke [78]	Breitenstein et al.; double-blind randomized placebo controlled study; n = 10, chronic stage, period 10 days [79] Leemann et al.; double-blind multiple case study, n = 12, subacute stage, dose 100 mg, period 2 weeks [80]	No/Not reported
PIRACETAM	Huber et al., Orgogozo et al.; post-hoc analysis of multicenter double-blind trial; n = 927; dose 12 g; period 8 weeks, acute stage [75,76] Enderby et al.; double-blind placebo-controlled randomized study, n = 158, period 12 weeks; acute stage [91] Kessler et al.; prospective, double-blind, placebo-controlled study, n = 24, dose 2,4 g, period 6 weeks; post-acute/chronic stage [77]	De Deyn et al.; multicenter double-blind trial; n = 927; dose 12 g; period 8 weeks, acute stage [74] Güngör et al.; randomized, double-blind study, n = 30, dose 4,8g, period 6 months, chronic stage [92]	Vertigo Sleep disturbances Tiredness Gastrointestinal irritability Seizures Agitation Vomiting Anxiety Nausea
FLUOXETINE		EFFECTS 2020; randomized, double-blind placebo controlled trial, n = 1500; dose 20 mg, period 6 months, acute stage [89]	Falls Bone fractures Epileptic seizures
BROMOCRIPTINE	Bragoni et al.; prospective, double-blind, placebo-controlled study; n = 11, chronic stage; dose: up to 30 mg; period 60 days [84]	Ashtary et al.; double-blind, placebo controlled study, n = 38, dose 10 mg, period 4 months, acute stage [83]	Atrial flutter and fibrillation Epileptic seizures Visual hallucinations Dystonic movements Tiredness Syncopal episode Nausea
GALANTAMINE	Hong et al.; prospective study; n = 45, dose up to 16 mg; period 12 weeks; chronic stage [93]		No
DESMOPRESIN	Tsikunov and Belekoskova; (intranasal usage); single cohort crossover design study, n = 26, chronic stage [94]		No
PROPRANOLOL	Beversdorf et al.; double-blind crossover study, dose 40 mg; chronic stage [95]		Not reported
tPA (TISSUE PLASMINOGEN ACTIVATOR)	Martins et al.; prospective study, n = 228; intravenous thrombolysis bolus dose depended on weight; acute stage [96]		
ATOMOXETINE	Yamada et al.; clinical study; n = 4, dose 120 mg, 3 weeks, subacute stages [97]		No
CITICOLINE	Alizadeh et al.; case report, 3 weeks, post-acute stage [98]		Not reported

**Table 4:** Summary of non-invasive brain stimulation in post-stroke aphasia (PSA).

Methods	Major Characteristics	Advantages	Limitations	
**rTMS** (repetitive transcranial magnetic stimulation)	Based on generating a magnetic pulse that induces power in various regions of the cerebral cortex. The rTMS procedure consists of regularly applied pulses of a given frequency within approximately 15 min. Depending on the frequency used, there is low-frequency stimulation (<1 Hz), which inhibits cortical excitability, and high-frequency stimulation (>1 Hz), which increases cortical excitability.	Balance the excitability of both hemispheres, as well as realign the linguistic network.	The need to establish the optimal treatment protocol and to take into account individual variability.	[100,101,102]
**tDCS** (transcranial direct current stimulation)	Non-invasive and safe therapeutic technique for stimulating the brain. This enables the polarization of the cell membranes of neurons, increasing or decreasing the level of cortical excitation. The nature of the induced cortical lesion depends on the electrode pole.	Normalization of brain activity promotes self-recovery.	The need for further clinical trials on a larger group of PSA.	[103]
